# A Scoping Review of Parenting Interventions with a Dual Focus on Improving Child Emotional and Physical Health

**DOI:** 10.21203/rs.3.rs-7150758/v1

**Published:** 2025-08-27

**Authors:** Daniel K. Cooper, Jayxa K. Alonzo, Francesca Lupini, Subina Saini, Nada M. Goodrum, Guillermo Wippold, Jada Mobley, Sarah R. Edmunds, Ronald Prinz, Michael Beets

**Affiliations:** University of South Carolina; University of South Carolina; University of South Carolina; University of South Carolina; University of South Carolina; University of South Carolina; University of South Carolina Upstate; University of South Carolina; University of South Carolina; University of South Carolina

## Abstract

**Background.:**

Children’s emotional and physical health are inextricably linked, as evidenced by the numerous shared health determinants known to impact each of these domains (e.g., adverse childhood experiences, supportive family relationships) as well as the bidirectional influence between them. As such, efforts have been made to intervene on these health determinants to jointly impact child emotional and physical health. However, this remains a nascent area of research, and little is known about the nature and impact of these interventions.

**Objective.:**

The present study aimed to identify what parenting interventions were available with a *dual focus* on improving child emotional and physical health. We examined the content of these interventions, how components were integrated, the findings for each health domain, and the extent to which health equity was considered.

**Design.:**

This scoping review was preregistered in PROSPERO (CRD42023369266) and followed PRISMA-ScR reporting guidelines for scoping reviews. We searched five online databases for articles published between 2012–2022 that (a) focused on children ages 2–12, (b) involved evaluating the effects of a parenting intervention, and (c) had a dual focus on promoting child emotional and physical health.

**Results:**

Out of the 2,065 records screened, thirty-one studies met inclusion criteria. Most interventions were intensive, delivered to parents only, and targeted high-risk families. While 59% demonstrated beneficial effects in both child health domains, findings were more consistent for emotional outcomes. Attention to health equity was limited, with few studies strategically recruiting minoritized populations, testing for subgroup differences in intervention effects, or applying culturally informed adaptations.

**Conclusions.:**

This review highlights a growing body of dual-focused parenting interventions with the potential to improve child health outcomes. However, gaps remain in terms of reach, scalability, integration of content, and equity. These findings can guide the development of holistic, accessible, and equity-focused interventions aimed at improving the health and well-being of children.

## Introduction

Child emotional and physical health are deeply interconnected, with an estimated 15% of children experiencing mental-physical multimorbidity (1) and extensive evidence linking emotional and physical health problems (2). In this review, emotional health refers to social, emotional, and cognitive-behavioral functioning, while physical health encompasses healthy lifestyle behaviors (e.g., sleep, eating habits, physical activity) and physical health-related indicators (e.g., weight/BMI, disease symptoms, somatic issues). These outcomes also share common social determinants, including neighborhood conditions, access to quality education and healthcare, structural racism, and supportive family relationships, that shape lifelong health trajectories (3, 4). Importantly, children from racially and ethnically minoritized backgrounds are disproportionately affected by these adverse social determinants, placing them at elevated risk for both emotional and physical health disparities (3, 5). Positive parenting practices represent one of the most powerful and modifiable levers for improving child well-being (6), making parenting interventions an important public health strategy. Programs that promote effective communication, positive reinforcement, limit setting, and consistent discipline have demonstrated success in reducing child behavioral problems while also fostering healthier lifestyle behaviors, such as improved sleep, eating habits, and physical activity (7–9). Systematic reviews have found that interventions incorporating parenting skills training can substantially impact both child social-behavioral-emotional outcomes (7, 8) and physical health (10, 11). Despite this evidence, most interventions have targeted either emotional or physical health in isolation, missing opportunities to address these interlinked domains simultaneously. *Dual*-*focused parenting interventions* that integrate both emotional and physical health promotion may be more effective, less burdensome for families, and especially valuable in addressing upstream drivers of health disparities. However, little is known about the scope, design, and impact of these programs. To address this gap, the present scoping review systematically examined dual-focused parenting interventions to assess the types of components used, outcomes targeted, and how equity considerations were incorporated, providing timely insights to guide more holistic and equitable approaches to child health promotion.

### Positive Parenting and Child Emotional and Physical Health

There is robust evidence that positive parenting, both as a naturally occurring behavior and as an intervention target, is associated with improved child emotional and physical health outcomes. Observational studies consistently demonstrate that higher levels of positive parenting (e.g., parental warmth, responsiveness, appropriate discipline) and lower levels of harsh parenting (e.g., yelling, spanking) are linked to greater child prosocial behavior, fewer internalizing and externalizing problems, and healthier lifestyle behaviors, including sleep, eating habits, and physical activity (12–14). For example, an international study of over 200,000 parents across 60 countries found that aggressive discipline practices (e.g., shouting, hitting) were associated with higher levels of child aggression and inattention, whereas non-aggressive discipline (e.g., reasoning) was linked to greater prosocial behavior (15). Similarly, systematic reviews have demonstrated that positive parenting practices are consistently associated with healthier child eating, physical activity, and sleep behaviors (16, 17). For example, studies have indicated that harsh or coercive parenting predicts poorer child eating habits and reduced sleep duration (18), while supportive parenting predicts greater physical activity and better dietary patterns (19).

Experimental studies further support the causal role of parenting in shaping child health outcomes. Meta-analyses of randomized controlled trials (RCTs) demonstrate that parenting interventions can effectively reduce harsh discipline and increase positive parenting behaviors, which in turn mediate improvements in children’s emotional and behavioral functioning (20, 21). For example, a meta-analysis of 14 RCTs found that reductions in harsh verbal and physical discipline following parenting interventions mediated decreases in child disruptive behavior (20). Though fewer studies have experimentally examined parenting behaviors and child physical health outcomes, available evidence suggests similar pathways. One study found that increases in lifestyle-related parenting practices following a parenting intervention mediated improvements in child physical activity among obese fathers and their children (22).

Despite this growing body of evidence, most parenting intervention research has remained siloed, focusing either on emotional or physical health outcomes, but rarely both. This remains the case even as the broader evidence base underscores the importance of addressing both domains in a unified approach. Widely disseminated parenting programs, such as Triple P and The Incredible Years, while highly effective in improving child emotional and behavioral outcomes, have traditionally given little focus to child physical health (8, 23). Similarly, many of the largest family-based physical health interventions, including landmark obesity prevention trials (24, 25), have largely neglected children’s emotional health. Nonetheless, evidence suggests that interventions targeting one domain may yield benefits in a domain that was not targeted (i.e., cross-over or ripple effects (26)). For example, a multisite evaluation of the Family Check-Up (FCU), an intervention originally designed to improve parenting and prevent conduct problems, demonstrated long-term positive effects on children’s diet and obesity outcomes from early childhood through middle childhood (27). Systematic reviews of lifestyle interventions in children and adults similarly report frequent cross-over effects on mental health and well-being (28, 29).

In recent years, there has been growing recognition of the potential to leverage positive parenting interventions to jointly promote children’s emotional and physical health. Emerging dual-focused models include adaptations of existing programs and novel interventions explicitly designed for this purpose (30–32). For example, Lifestyle Triple P, an adaptation that directs parenting skills typically applied to child behavioral and emotional difficulties to the promotion of child nutrition and physical activity, has shown promising effects on child BMI and related behaviors (32). Similarly, the FCU has been enhanced to incorporate obesity prevention content for young children from racially and economically diverse backgrounds, yielding positive effects on both emotional regulation (33, 34) and physical health outcomes (e.g., child health behaviors, BMI). Despite these advances, no comprehensive review has synthesized the current landscape of dual-focus parenting interventions. A systematic understanding of this growing field is critical for informing future intervention development, implementation, and dissemination.

### Equity-Focused Approaches to Child Health Promotion

Given the substantial health inequities and structural barriers faced by minoritized populations, there is increasing recognition that child health promotion efforts must incorporate a more deliberate and substantive focus on equity (35–37). The main driver of these health inequities is structural racism, a system of policies, practices, and cultural norms that collectively reinforce one another to sustain racial inequality (3). This has led to unequal distribution of resources and differences in exposure to social determinants of health, such as access to safe neighborhoods, affordable housing, quality education, healthcare, and healthy food. Such inequitable environmental conditions, created and maintained by structural racism, are shown to be detrimental to public health (4) and have been likened to swimming in toxic waters (37). As public health researchers and practitioners, our role is to help detoxify these waters by creating new streams, tides, and currents that dismantle structural racism and move towards health equity.

While there is growing momentum toward equity in child health promotion, health equity frameworks have been underutilized in dual-focused parenting interventions that target both emotional and physical health. This is a critical gap, given that structural inequities often operate across and between these domains. For example, chronic stress related to racism and poverty can simultaneously disrupt emotional regulation and sleep, while neighborhood conditions may limit both opportunities for physical activity and access to culturally relevant mental health supports (3, 4). Dual-focused interventions are uniquely positioned to address the intersections of these challenges, but only if equity is at the center of their design and evaluation. Yet, a systematic review of 240 programs listed in the Blueprints for Healthy Youth Development online clearinghouse found that most did not qualify as equity-promoting interventions (35). For example, only 31% of programs were culturally tailored and just 19% tested for subgroup differences in intervention effects. Insufficient attention to equity in program design, delivery, and evaluation represents an important limitation. To help address this issue, the present review expanded on the criteria outlined by Buckley et al. to assess how equity considerations have been incorporated into dual-focused parenting interventions aimed at improving child emotional and physical health.

### Present Study

Given the increasingly recognized importance of integrated, family-based approaches to child health, there is a need for a comprehensive review that evaluates interventions targeting both emotional and physical health outcomes in children. The present scoping review addressed this need by systematically assessing the extent and nature of the available research on parenting interventions with a dual focus on child emotional and physical health. The objectives of this scoping review were to synthesize and describe: (a) the intervention characteristics and components; (b) the findings related to child emotional and physical health; and (c) the extent to which interventions incorporated a focus on health equity. Generating a clearer understanding of the current literature is critical to guiding the development of future intervention strategies that are both holistic and equity-focused in promoting child health.

## Methods

### Protocol and Registration

To accomplish these aims, we conducted a scoping review following the Preferred Reporting Items for Systematic Reviews and Meta-Analyses (PRISMA) 2020 guidelines (38) and the PRISMA extension for Scoping Reviews (PRISMA-ScR) (39). It was also informed by Tawfik et al.’s (40) scoping review methodology. The protocol was preregistered in PROSPERO (CRD42023369266) on March 18, 2023. The methods used to identify, select, and analyze relevant studies are detailed below.

### Eligibility Criteria

Eligibility criteria were developed using the PICO (Population, Intervention, Comparison, and Outcome) framework (41). Studies were included if they met the following criteria: (a) included children ages 2–12; (b) the intervention was a parenting program delivered directly to parents, with explicit instruction in positive parenting skills (e.g., communication, problem-solving, positive reinforcement, limit setting, effective discipline, or strategies for effective play); (c) included at least one pre- and post-intervention measure of child emotional health (defined as social, emotional, or cognitive-behavioral functioning); (d) included at least one pre-post measure of child physical health (defined as healthy lifestyle behaviors or physical health-related indicators); and (e) included peer-reviewed articles, preprints, or dissertations published in English between 2013 and 2023.

We focused on the 2 to 12 age range because this period represents a phase of parenting during which parents serve as the primary socialization agents, and positive parenting practices are central to shaping both emotional and physical health outcomes (13, 42). Parenting skills and strategies during this phase differ considerably from those used with infants (which are more focused on attachment and basic care) (43) or with adolescents (which increasingly emphasize autonomy and peer relationships) (44), and many positive parenting intervention studies target families within this age range (8). To meet our definition of a parenting intervention, programs were required to provide more than simply parent involvement or parent education. Specifically, interventions needed to offer active training or skill development in core positive parenting practices (specified above) that promote child emotional and physical well-being. Studies that did not meet these criteria were excluded from the review.

### Search Strategy

We conducted comprehensive searches across the following electronic databases: PubMed, PsychINFO, CINAHL, Embase, and Web of Science (grey literature), which included Science Citation Index Expanded (SCI-EXPANDED), Social Sciences Citation Index (SSCI), Arts & Humanities Citation Index (A&HCI), Emerging Sources Citation Index (ESCI), Current Chemical Reactions (CCR-EXPANDED), and Index Chemicus (IC). The search strategy was designed to identify studies focused on the target population (children ages 2–12 years), interventions (parent training programs), and outcomes (child emotional and physical health-related indicators).

An iterative search development process was used in collaboration with a University Librarian and members of the research team. Initial search terms were drafted by the first author and peer-reviewed by three study team members using the *Evidence*-*Based Checklist for the Peer Review of Electronic Search Strategies* (PRESS EBC) (45). The search strategy was then tested and verified by two additional team members to confirm the accuracy and reproducibility of results (see Supplemental File 1 for the full search string for PubMed). These search terms were then adapted to fit the format of the other databases. Search results from each database were exported as RIS files and imported to Covidence, where duplicates were automatically identified and removed prior to screening. All database searches were completed in March and April 2023. The review was limited to peer-reviewed articles and grey literature (i.e., preprints and dissertations). Reference lists and clinical trials registry numbers of included articles were screened to identify additional relevant studies.

### Study Selection Process

Articles were selected using a two-step screening process conducted by two independent, blinded reviewers. In the first phase, titles and abstracts were screened to assess potential eligibility for full-text review. Prior to initiating full screening, the team piloted the process on a random sample of 10 articles to ensure consistency in applying eligibility criteria. Any discrepancies were discussed, and screening procedures were refined accordingly. After piloting, the two reviewers independently screened all titles and abstracts, with conflicts resolved through discussion and consensus.

In the second phase, the same two reviewers independently assessed the full texts of all articles identified as potentially eligible. As with title and abstract screening, discrepancies in full-text decisions were reconciled through discussion until consensus was reached.

**Figure 1:**
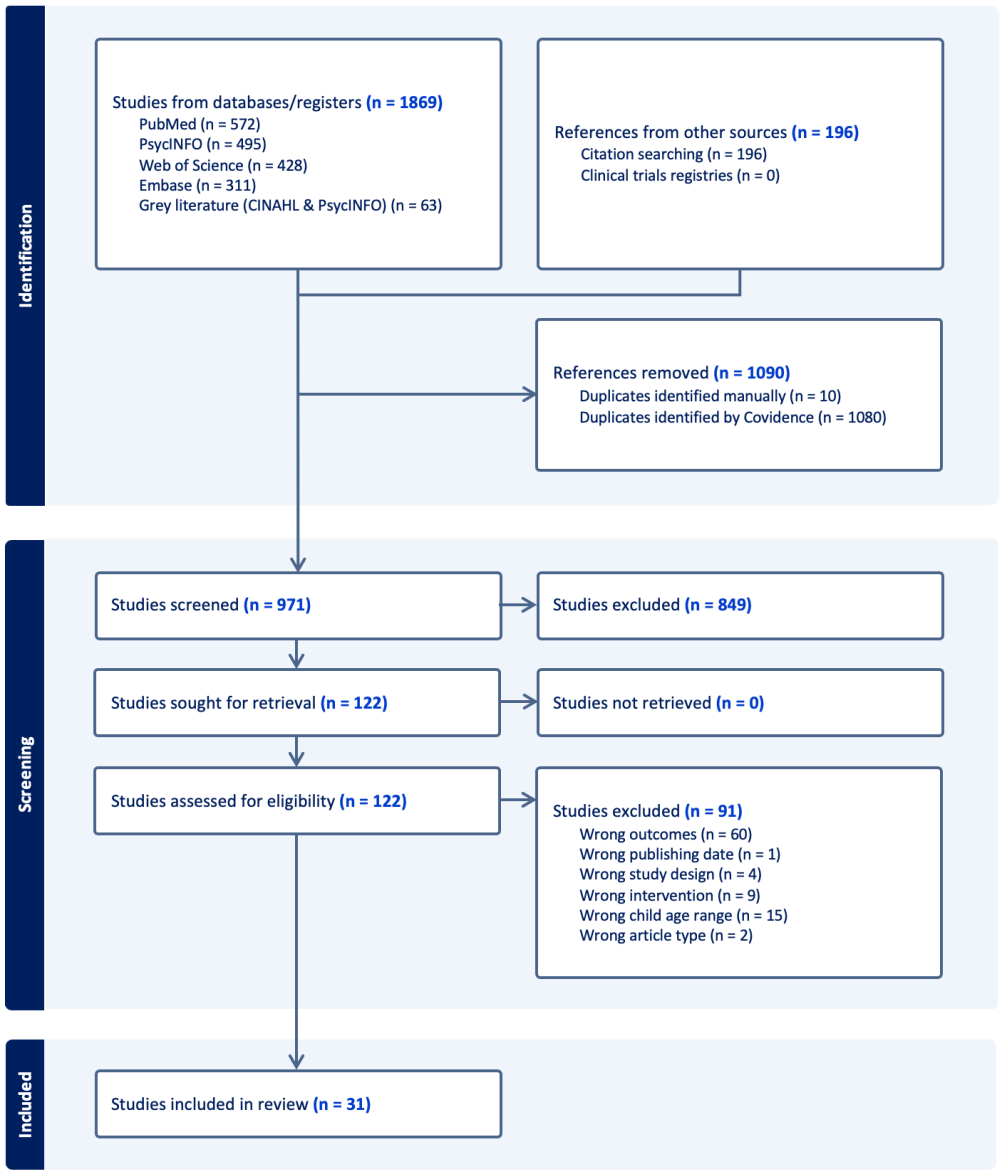
PRISMA Flow Diagram *Note*. The term “studies” in this figure refers to the number of included articles, which may not correspond to the number of unique interventions. In some cases, multiple articles report on the same intervention or study. The final 31 studies included in our review described 29 unique interventions.

To enhance completeness, we conducted two additional steps: (1) searches of clinical trials registries to identify reports of relevant intervention outcomes not captured in the initial database search, and (2) hand-searching the reference lists of included articles to identify additional eligible studies (or to find articles that included a greater description of the intervention components). The full selection process is illustrated in the PRISMA flow diagram (Fig. 1).

### Data Extraction

Articles were divided between four team members, with the first author reviewing all included articles, and the other three team members serving as the second reviewer (each reviewing one-third of the articles). Data were independently extracted using both the Covidence Extraction Tool and standardized Excel spreadsheets. The extraction process was guided by the PICO framework and included the following domains: participant and study characteristics (e.g., participant demographics, study country, study design, number of intervention arms), intervention characteristics (e.g., number and length of sessions, delivery setting and format, intervention components), and study outcomes (pre-post changes in child emotional and physical health).

Additionally, the team extracted information related to the application of a health equity lens, based on an expanded version of the criteria outlined by Buckley et al. (35). Specifically, we coded whether each study: (a) reported the sociocultural characteristics of the sample (e.g., ethnicity/race, nativity, socioeconomic status, sexual or gender identity); (b) employed purposeful recruitment efforts to engage minoritized groups (e.g., explicitly reported a specific strategy for enhancing the sociocultural diversity of their sample); (c) tested for subgroup differences in intervention effects across minoritized groups (e.g., compared intervention effects across racial and ethnic groups); (d) stated explicit health equity objectives; and (e) culturally adapted or tailored the intervention. All extracted data were reviewed for accuracy and consistency, with extraction dyads reaching consensus on each article prior to finalizing the data. The resulting data charting form is available in Supplemental File 2.

### Data Synthesis

After data extraction was complete, we conducted a descriptive synthesis of the included studies, focusing on key study and participant information, intervention characteristics and components, and study findings. As a first step, we generated a summary of basic study characteristics and participant demographics by calculating pooled means across studies (full details about these calculations are provided in Table 1 description). Studies were then categorized by core intervention features, including delivery length and format (e.g., group-based, individual, hybrid), setting (e.g., community, school, clinic, home, virtual or in-person), and intervention components (e.g., positive parenting skills, health education, and child components). Next, studies were classified based on the degree to which they applied a health equity lens using the aforementioned criteria.

Intervention outcomes were synthesized and categorized by domain: (a) child emotional health (i.e., social, emotional, and cognitive-behavioral functioning) and (b) child physical health, including healthy lifestyle behaviors (i.e., sleep, eating habits, physical activity, and screen time) and broader physical health indicators (i.e., weight, BMI, disease symptom management, and somatic concerns). Intervention effects were classified as beneficial (i.e., significant improvements in the intended direction), adverse (i.e., significant iatrogenic effects), or null (i.e., no significant effects), based on a significance level of *p* < .05. When available, group × time interactions were prioritized for evaluating outcomes. In addition, studies were coded according to their breadth of impact: those demonstrating improvements in one domain were labeled as having a “single effect,” those showing improvements in both domains as having a “dual effect,” and those with no improvements as having “no effect.”

As this is a scoping review, no formal meta-analysis was conducted. Instead, the data synthesis followed an iterative process in which the first author and study team developed and refined categories to best capture the patterns of intervention content and outcomes contained in the literature. Findings are summarized descriptively and presented in Tables 1–5 to support transparency and replicability.

## Results

### Selection of Results

The initial search yielded 2,065 records: 1,869 from the database search and 196 from the reference lists of included articles. After duplicate removal, 971 unique records remained. Title and abstract screening (*N* = 971) was conducted in Covidence by two independent reviewers. Full-text review was completed for 122 articles. Any discrepancies in coding (e.g., discordant inclusion/exclusion decisions) were resolved through consensus discussions.

The most common reasons for exclusion at the full-text stage were: (1) not reporting pre-post measures of both child emotional and physical health outcomes; (2) not targeting the specified child age range (2–12 years); or (3) the intervention not meeting inclusion criteria for parent training (e.g., school-based program only, child-only intervention). Following this process, 31 articles representing 29 unique interventions met full eligibility criteria and were included in the final synthesis (see Figure 1 for PRISMA flow diagram).

### Study and Participant Characteristics

Table 1 summarizes the key characteristics of the 31 included studies. Most interventions were conducted in the United States (*n* = 20, 65%), with the remaining studies based in Europe (Denmark, the Netherlands, Finland, Sweden, Germany, and Spain), Australia, Malaysia, and Brazil. Studies employed a range of research designs, including randomized controlled trials (RCTs; *n* = 19, 61%), prospective cohort studies (*n* = 9, 29%), and case studies (studies with less than 5 participants; *n* = 3, 10%). Samples ranged from one to 697 participants, with a pooled average sample size of 109. Across the studies, mothers were overrepresented, with an average of 86% of participants being mothers. No included study reported a sample with greater than 50% father participation.

Participant demographics varied considerably across studies. While a portion of studies recruited racially and ethnically diverse samples, the majority included predominantly White, non-Hispanic families. Only three studies included samples in which over 50% of participants identified as members of racially or ethnically minoritized groups. Across all studies, households tended to be middle or high-income, two-parent (77%), and highly educated (58%).

### Intervention Characteristics

The 29 unique parenting interventions included in this review were highly variable in their focus, format, and delivery. Most studies focused on the prevention or treatment of specific child behavioral or emotional difficulties (e.g., externalizing problems, anxiety, ASD-related behaviors) or child health problems (e.g., overweight/obesity). Only a minority (*n* = 3, 10%) used a universal prevention approach aimed at promoting general child well-being. Intervention settings varied widely: over one third were delivered in medical settings (e.g., hospitals, pediatric clinics; *n* = 10, 34%), with others conducted in clinical mental health settings (*n* = 7, 24%), or directly in the home (*n* = 4, 14%). Increasingly, interventions incorporated online delivery or flexible delivery formats, either as stand-alone digital programs (*n* = 4, 14%) or as blended models combining two or more formats, such as offering phone or online sessions paired with in-person sessions (*n* = 11, 38%).

Intervention formats ranged from brief, single-session workshops (46) to multi-session, manualized programs delivered over several months (47), with session frequency typically weekly or biweekly. Lumeng et al.’s intervention was the longest, with 14 parenting sessions and over 60 school-based child lessons (48). Theoretical foundations were diverse but often drew from well-established frameworks, including social learning theory, cognitive-behavioral principles, attachment theory, and family systems models. Only 41% (*n* = 12) of the studies reported their guiding intervention theory. However, nearly all studies described using strategies that aligned with social learning theory (49) or operant learning theory (50), which aim to interrupt conflictual patterns of interaction using behavioral reinforcements and consequences.

Interventionists most often included highly trained master’s or doctoral-level professionals (e.g., psychologists, nurses, social workers). Table 2 provides an overview of key intervention components.

Additionally, we assessed programs for their incorporation of a health equity lens, building on Buckley et al. (35). Overall, few studies had a strong focus on health equity based on our evaluation criteria. Although many studies reported on one or more sociocultural characteristics of their sample (e.g., ethnicity-race, income/SES), few studies used purposeful recruiting methods (*n* = 5, 19%), tested for subgroup differences in intervention effects (*n* = 2, 8%), had explicit health equity objectives (*n* = 5, 19%), or used a culturally tailored (or adapted) interventions (*n* = 3, 12%).

### Intervention Components

To synthesize the components of the parenting programs, we classified each program according to its inclusion of core positive parenting strategies and parent-directed information related to promoting their children’s health (see Table 3). Nearly all interventions focused on enhancing positive parenting skills shown to improve parent-child relationships and support healthy child development. The most commonly included strategies were limit setting (*n* = 26, 90%), positive reinforcement (*n* = 25, 86%), and communication skills (*n* = 24, 84%), reflecting a strong emphasis on helping parents establish clear expectations and boundaries, reinforce positive behaviors, and foster open communication with their children. These strategies were often applied to promoting both child emotional and physical health outcomes. In contrast, strategies for effective play (an important tool for promoting positive interactions and developmental gains) were included in only 32% of interventions.

In terms of physical health-related content, a majority of interventions incorporated components targeting children’s nutrition and eating habits (*n* = 18, 62%) and sleep behaviors (*n* = 16, 55%). Fewer interventions addressed other important areas of child physical health, such as physical activity (*n* = 11, 38%) or reducing screen time (*n* = 11, 38%). That said, most studies (*n* = 18, 62%) targeted multiple healthy lifestyle behaviors within the same intervention and two studies targeted all four lifestyle behaviors in their intervention (i.e., *FCU4Health* and *Grow*).

Furthermore, while all included interventions engaged parents directly, only 31% of programs also involved a child-focused component. The limited inclusion of direct child engagement suggests that most parenting interventions rely primarily on parent behavior change as the mechanism for influencing child outcomes, rather than combining parent and child involvement.

### Intervention Outcomes

To synthesize intervention outcomes, we organized effects based on key child outcome domains: emotional health, healthy lifestyle behaviors, and health-related indicators (see Table 4). Our aim was to explore whether certain outcomes were more readily impacted by parenting interventions, and to assess whether interventions consistently achieved improvements across both child health domains.

Reassuringly, none of the included studies reported adverse effects on any child emotional or physical health outcomes. However, many studies reported a mix of beneficial and null findings across domains. For child emotional health, approximately half of reported effects were null, while the other half demonstrated statistically significant improvements in the intended direction. In contrast, fewer beneficial effects were observed for child physical health outcomes, especially physical health-related indicators, with most results (58%) in this domain being null. Weight or BMI had the fewest positive effects, with 92% of findings being null.

When we examined the overall pattern of effects across studies, a majority (*n* = 16, 59%) demonstrated dual effects, meaning they reported at least one significant improvement in each of the two child health domains. Another subset of studies (*n* = 6, 22%) achieved significant improvements in only one domain, either emotional or physical health. The remaining 19% of studies did not report significant effects in either domain. As illustrated in Table 5, several studies achieved dual effects despite having a preponderance of null findings within one or both domains. Exploratory comparisons suggest that studies classified as having dual effects tended to report a greater number of outcomes overall, increasing the likelihood of identifying at least one positive effect per domain. For example, Malow et al. (65) reported on 21 child health outcomes and found dual effects, whereas Raby (68) reported on five child health outcomes and did not find effects on either domain. This pattern highlights the need for caution when interpreting these results and suggests that future meta-analyses should account for the number of outcomes reported to avoid bias in estimating intervention effectiveness.

## Discussion

The purpose of this study was to conduct a scoping review of parenting interventions with a dual focus on promoting children’s emotional and physical health. Specifically, we aimed to (a) describe the intervention characteristics and components; (b) synthesize findings on child emotional and physical health outcomes; and (c) assess the extent to which interventions addressed health equity. This review fills an important gap in the literature, as no prior efforts have systematically mapped the landscape of dual-focused parenting interventions. A systematic understanding of this growing field is critical for informing future intervention development, implementation, and dissemination. Key findings highlight the predominance of intensive, clinically delivered programs targeting high-risk populations, the more consistent impacts of interventions on emotional versus physical health outcomes, the limited integration of both domains within many interventions, and the minimal attention to health equity, particularly with respect to cultural tailoring. These findings are presented in the order of our study objectives.

### Intervention Characteristics and Components (Objective 1)

Most of the 29 included interventions were quite intensive, with many requiring over 10 sessions and combining in-person and phone-based delivery. Notably, the majority were designed to treat existing behavioral or health problems in children already experiencing significant issues rather than to serve as universal prevention programs. This represents a critical gap in the literature. Scalable, low-cost parenting support, particularly universal approaches, are urgently needed to achieve population-level improvements in child well-being and interrupt adverse health trajectories early in development (77).

Encouragingly, interventions were delivered across a range of contexts and formats, demonstrating flexible dissemination potential. However, most programs were facilitated by highly trained clinicians (master’s or doctoral level), with few studies utilizing paraprofessionals or community health workers. This reliance on specialist providers may limit the reach and long-term sustainability of these programs, especially in under-resourced communities.

In terms of content, the majority of interventions emphasized core positive parenting skills such as limit setting, positive reinforcement, and effective communication. These strategies were applied to promoting both child emotional and physical health outcomes. Fewer programs incorporated strategies for effective parent-child play or content aimed at improving parents’ own well-being, despite evidence that parent mental health (e.g., depression, anxiety) is directly related to child emotional and behavioral outcomes (78) and may bolster intervention effects (79). Additionally, only a minority of interventions (31%) directly engaged children alongside parents, suggesting that most programs relied on parent behavior change alone to influence child outcomes. While this reflects common practice, future research should explore the added value of child involvement for maximizing intervention effects.

Another important finding was the consistent underrepresentation of fathers. Across studies, mothers were overwhelmingly the primary participants. This trend aligns with previous reviews of parenting interventions and reflects broader recruitment and engagement challenges in parenting research (80, 81). Given robust evidence that fathers play a crucial role in shaping child development, including emotion regulation, behavior, and physical health outcomes (80), future programs should make intentional efforts to engage fathers and test whether outcomes differ based on caregiver involvement.

### Intervention Outcomes on Child Emotional and Physical Health (Objective 2)

We synthesized intervention effects across 264 child outcomes. While fewer than half of reported effects were statistically significant, a majority of interventions demonstrated at least some positive impact. Overall, parenting programs demonstrated more consistent positive impacts on child emotional health than on physical health outcomes. Among emotional outcomes, the greatest effects were observed for the cognitive-behavioral functioning subdomain (i.e., 56% of observed effects were positive). For physical health, improvements were more modest, with the greatest positive effects seen in child sleep; fewer effects were found for eating habits, physical activity or BMI-related indicators. Nonetheless, 59% of interventions produced “dual effects,” or improvement on both health domains, underscoring the potential of positive parenting programs to promote integrated child well-being. These findings are particularly striking given that many parenting interventions have historically focused on a single domain, and few prior reviews have examined cross-over effects (26, 29). It is worth noting, however, that our synthesis may overemphasize the presence of intervention effects, as studies reporting a large number of outcomes were more likely to report at least one significant finding in each domain.

That said, null findings were common, particularly for physical health outcomes. Notably, a substantial proportion (92%) of the null findings for physical health outcomes pertained to BMI, a metric that is historically difficult to influence through intervention (24, 25). Considering BMI’s limited sensitivity to change, future research should exercise caution in using it as the main indicator of physical health and instead consider including alternative or additional health metrics. A second potential explanation is that many interventions lacked a strong and balanced dual focus. Rather than fully integrating components that target both emotional and physical health, many programs emphasized one domain over the other. For example, the Strongest Families Smart Website intervention (74) incorporated numerous positive parenting strategies aimed at improving children’s emotional and behavioral functioning but contained no explicit content addressing child physical health. Conversely, the ENTREN-F program (72) focused heavily on promoting healthy lifestyle behaviors, such as diet and physical activity, yet offered limited content on using positive parenting strategies to impact child social or emotional health. This imbalance in focus may reduce the likelihood of producing robust, cross-domain effects. In contrast, interventions with a more integrated approach, such as FCU4Health (33, 34), appear better positioned to impact both domains. FCU4Health included a strong foundation in positive parenting skills, coupled with actionable strategies for improving child sleep hygiene, limiting unhealthy snacking, and managing screen use. Notably, it demonstrated positive effects across both emotional and physical health outcomes, illustrating the potential of fully dual-focused models.

A third factor contributing to the inconsistent findings may be the substantial variability in intervention content across studies. The breadth of components included in the programs makes it challenging to tease apart which ingredients are most effective in improving specific emotional and physical health outcomes. Although bundling components into comprehensive intervention packages is supported by prior research (82), further studies are needed to determine the unique and additive effects of individual intervention components. Future research employing meta-analytic techniques or experimental designs (e.g., factorial or SMART designs) rooted in optimization frameworks may be especially valuable for identifying which intervention components or combinations produce the strongest effects in each health domain (83). Such work could advance the precision and efficiency of parenting interventions targeting both emotional and physical health.

### Incorporation of a Health Equity Lens (Objective 3)

The present review revealed minimal attention to health equity across included studies. Most samples were predominantly White, middle-class, highly educated, and conducted in high-income countries, particularly the U.S. and Europe. Although nearly all studies reported basic sociodemographic characteristics, few went further to strategically recruit minoritized populations, test for subgroup differences, or apply culturally informed adaptations or frameworks. This lack of equity integration mirrors broader gaps highlighted in prior reviews of health promotion interventions (35). Overlooking equity principles may constrain both the generalizability and effectiveness of dual-focused parenting programs, especially for families disproportionately impacted by structural disadvantage (84).

That said, a small number of studies offer promising models for equity-focused design. For example, the enhanced ParentCorps intervention was co-developed with community partners to ensure alignment with the cultural values, lived experiences, and contextual stressors facing lowincome families of diverse racial backgrounds (47). This partnership-driven approach helped to foster trust, ensure relevance, and increase engagement in a historically underserved population. Similarly, the FCU4Health intervention (33, 34) applied a cultural tailoring framework that emphasized flexible, values-based delivery rather than broad, uniform adaptations. Rather than presuming cultural homogeneity within racialized groups, the FCU4Health model emphasized shared, cross-cultural human values (e.g., the importance of family, storytelling, and collaboration) and allowed for cultural tailoring within sessions. Facilitators were encouraged to address the broader structural and environmental contexts contributing to child health challenges (e.g., poverty, discrimination), lending to a more holistic, equity-informed approach. These two exemplars underscore the feasibility and importance of embedding cultural responsiveness into dual-focused parenting programs. Future research should prioritize participatory design approaches, test equity-focused mechanisms of change, and examine how cultural tailoring strategies influence program engagement, fidelity, and outcomes across diverse family systems.

### Strengths and Limitations

This review is strengthened by rigorous methods, including pre-registration, peer-reviewed search strategies, blinded screening and extraction by multiple coders, and supplementary searches of trial registries and reference lists to maximize completeness. The review also extends prior work by systematically evaluating health equity considerations. However, several limitations should be acknowledged. First, we did not conduct a formal risk of bias assessment. While this is a common omission in scoping reviews, it is important for interpreting the strength of available evidence once a body of literature becomes more substantial (39). Second, extraction of intervention components relied solely on published reports or linked citations. Some programs may include additional elements not fully described in our review of the literature. Third, variation in outcome reporting across studies, particularly outliers with very large numbers of reported outcomes, may have influenced our synthesis of effects. Future reviews could address this by the use of weighting or by conducting sensitivity analyses. Fourth, we were not able to extract consistent information about the designation of primary versus secondary outcomes, which limited our ability to interpret the strength of each study’s dual focus. Fifth, we were also unable to systematically account for differences in how each outcome was measured (e.g., use of different measurement tools or reporters), which limited our ability to assess the robustness of the findings across emotional and physical health outcomes.

### Implications

This scoping review provides new insights into the state of dual-focused parenting interventions designed to promote children’s emotional and physical health. Although these interventions are relatively scant, the growing body of literature suggests that parenting-focused approaches can influence both child domains, offering a promising public health strategy to address complex, co-occurring health needs in children. Given the rising rates of childhood mental health concerns, obesity, and other challenging conditions (85) (which are often exacerbated by structural inequities (3)), investing in parenting interventions that build skills and protective family processes could help interrupt adverse health trajectories. Moreover, supporting parents through scalable interventions may complement broader public health and policy initiatives by addressing upstream drivers of health disparities (e.g., (86)). Successful examples of this approach already exist. For instance, state-level partnerships to embed evidence-based parenting programs, like the Family Check-Up, into primary care settings show promise in expanding access to quality parenting supports for families facing economic disadvantages (87, 88).

Future research should aim to expand this field in several important ways. First, there is a need to develop more brief, universally delivered interventions that can reach families across the population, not just those with identified risks, given that many existing interventions are lengthy (10–20 sessions) and narrowly targeted (focused on a specific disorder). Second, advancing the science of intervention optimization will be valuable. Research that identifies which parenting components are most effective for improving different child health outcomes would help strengthen intervention design. Approaches such as the Multiphase Optimization Strategy (MOST) (83) could provide a useful framework for refining dual-focused interventions and addressing the high proportion of null effects observed in the current literature. Building this knowledge base is critical for informing the next generation of parenting programs. Ultimately, integrating dual-focused parenting supports into public health infrastructure could help address pressing child health crises and advance health equity on a population scale.

## Supplementary Files

This is a list of supplementary files associated with this preprint. Click to download.

- SupplementalFile1PubMedSearchStrategy.docx
- SupplementalFile2ExtractionFormTemplate.xlsx

## Figures and Tables

**Table 1:** Study and Participant Characteristics

Study Characteristics	Study Totals	
	Pooled Means	Number of studies
Study country^a^		
US	65%	20
Outside US	39%	12
Study design		
RCT (RCT or cluster RCT)	61%	19
Prospective cohort study	29%	9
Case study	10%	3
Mean sample size	109 participants	31
Range	1–697 participants	
Mean child age	6.6 years	27
Child gender (mean % girls)	39%	26
Child ethnic-racial background		
Black or African American	11%	10
Hispanic or Latine	20%	11
Asian or Asian American	16%	8
Indigenous or Native American	2%	4
White	72%	15
Mean parent age	36.8 years	11
Parent gender (% mothers)	86% mothers	11
Parent education		
High school or less	31%	17
More than high school	58%	17
Family structure		
Two-parent home	77%	10
Single-parent home	18%	9

*Note*. Study totals were calculated based on data provided in the included studies. Due to inconsistent reporting, many categories had substantial missing data. Pooled means = child and parent characteristics were calculated as unweighted pooled means. Number of studies = the number of studies that reported data in this category (e.g., 10 studies reported the percentage of Black or African American children in their sample). Parent education = High school or less included parents with a high school degree or equivalent (e.g., GED, completed secondary education); more than high school included parents who completed some college, had college degrees or higher. Family structure = two-parent home included parents who were married or reported having two parents or caregivers living in the home; single-parent home included families with one parent living in the home.

a Study country does not add up to 100% because one study was conducted in multiple countries and therefore counted in both category options.

**Table 2:** Intervention Characteristics (N = 29)

Intervention name (citation)	Target intervention population	Setting of intervention	Who takes part in intervention	Who delivers intervention	Group or individual	Length of intervention	intervention Theory	Attention to Equity
I-InTERACT Express (51)	TBI	virtual through hospital settings	parents	clinical psychology grad students or licensed psychologists	individual	7 40–60 min sessions (plus 1 booster)	not reported	1, 3, and 4
RUPP (52)	ASD	in-home and telephone	parents	masters-level therapists	individual	11–13 60–90 min sessions + 3 booster sessions	behavioral analytic orientation	1
FCU4Health (33,34)	overweight/obesity	in clinic (primary care), at home, or other private location	parents	trained FCU4Health coordinators	individual	dosage range: 1.40719.2 hours, average dosage: 53.79 hours	not reported	1, 2, 3, and 4
Grow (53)	universal focus	child development center or a local YMCA	parents	certified facilitators	group	5 90-min sessions	social cognitive theory, positive youth development	1
Parent training intervention (54)	primary anxiety disorder and chronic insomnia	university mental health center	parents	primary investigator	individual	4 90 mins sessions + 4 30-min phone check-ins	not reported	1
Family-based telemedicine intervention (55)	overweight/obesity	virtual via telemedicine	parents and child	grad students or licensed psychologists	groups	14 60-min sessions	not reported	1
Enhanced ParentCorps (47)	children at risk for obesity	public elementary schools	parents and child	mental health professionals, teachers and ed assistants	group	14 120-min sessions	family-centered “whole child” approach	1, 2, 4, and 5
PCIT-Health (56)	children at risk for obesity	virtual-telehealth	Parents and child	clinical psychology PhD students	individual	12 sessions (length of sessions not reported)	not reported	1* (case study)
More and Less parent group program (57)	overweight/obesity	outpatient paediatric clinics	parents	trained dieticians	group	10 90-min sessions	not reported	1
LEAP (58)	ADHD and physical activity issues	hospital outpatient clinic or virtual	parents	licensed psychologists	group	8–9 90-min sessions	not reported	1
Behavioral sleep intervention (59)	ADHD and a parent with sleep problems	paediatrician’s office, the hospital clinic, or home	parents	licensed psychologists	individual	2 30–60 min sessions + 1 follow-up phone call	not reported	1
PT-F (60,61)	ASD and feeding problems	mental health clinic	parents	doctoral or masters level therapists	individual	Pilot: 9 60–90 min sessions RCT: 11 60–90 min sessions + 3 virtual parent coaching sessions	not reported	1
Mindful M&M’s (62)	disruptive behavior disorder	university mental health clinic	parents and child	trained practitioner	individual	4 60 min sessions of parent training + 4 60 min sessions of child mindfulness training	not reported	0* (case study)
parent training program (63)	ASD	not reported	parents	primary investigator	group and individual	2 sessions (length of sessions not reported)	not reported	1 and 5
Combined Sleep and Standard Behavioral Treatment (64)	emotional and/or behavioral problems	behavioral health clinic	parents and child	doctoral-level psychologists	individual	3–17 sessions (session length not reported)	sleep deficits degrade cognitive functioning and worsen emotion regulation due to sleep’s effects on the prefrontal cortex	1
HS+POPS+IYS (48)	universal (within Head Start)	in-home and school setting	Parent and child	master’s-level mental health specialist	group and individual	POPS: 6 child lessons and 8 75 min parent lessons; IYS: 60 child sessions and 10–14 parent training sessions	social cognitive theory	1, 2, and 4
Parent-based sleep education (65)	ASD and sleep difficulties	medical centers	parents	trained educators	individual or group	1 60-min session or 2 120-min sessions + 2 brief phone calls	not reported	1
Parent Management Training + Sleep Train Program (66)	behavioral problems	university mental health clinic	parents	doctoral student therapists	individual	14 60-min sessions	not reported	1
Hassle Free Mealtimes Triple P (67)	feeding or mealtime difficulties	not reported	parents	psychologists	group	1 120-min session	principles of behavioral family intervention and social learning	1
Girls Growing in Wellness and Balance (68)	universal delivery	middle school	parents and child	school psychologist	group	4 90-min sessions	Maudsley method; Minuchin Family Therapy	1
CBT-based Bibliotherapy Plus Doll (69)	nighttime fears and co-sleeping problems	clinical setting	parents	doctoral level therapist	individual	1 60-min session + 4 10–20 min phone sessions	CBT	1, 4
Behavioral parent training (70)	ASD and challenging behavior	university campus	parents	master’s clinician	group	6 120-min sessions	not reported	1
DELFIN parenting program (71)	type 1 diabetes	children’s hospital	parents	psychologist	group	5 120-min sessions + 1 phone call	CBT parent training	1
ENTREN-F program (72)	overweight/obesity	primary care	parents and child	research team members	groups	6 120-min parent sessions + 9 120-min child sessions + 3 120-min joint parent-child sessions	cognitive behavioral perspective	0
Cognitive-behavioral, skill-building intervention (73)	overweight/obesity	pediatric primary care	parents	trained interventionists	individual	4 20–45 min sessions	cognitive-behavioral; information, motivation, and behavior skills (IMB) framework	1 and 2
SFSW intervention (74)	behavioral problems	self-directed online sessions + telephone	parents	licensed health care professionals	individual	11 selfdirected sessions + 45-min phone coaching sessions + 1 booster session	not reported	1 and 5
Parent-Assisted CBT (75)	sleep problems and nighttime fears	not reported	parents and child	not reported	individual	5 75-min sessions	CBT	1* (case study)
CHIP-Family^a^ (46)	congenital heart disease	not reported	parents, child, and sibling or friend	licensed clinical and health psychologists	group and individual	1 6-hour workshop + 1 60-min individual session	not reported	1
Triple P (76)	type 1 diabetes	children’s hospital	parents	clinical psychologist	individual	10 60-min sessions	not reported	1 and 2

*Note*. Table includes information from all 31 included articles for a total of 29 unique interventions. *Attention to Equity*: 0 = none reported. 1 = reported sociocultural characteristics of sample (e.g., ethnicity-race, SES, SGM, immigration status, disabilities). 2 = purposeful recruiting: explicit effort to increase the sociocultural diversity of sample. 3 = testing subgroup differences, such as differential intervention effects for minoritized groups (ethnically-racially minoritized, immigrants, economically disadvantaged, or SGM). 4 = health equity focused study objectives, such as the goal of the study was to improve access or quality of care for minoritized groups. 5 = cultural adaptations: intervention materials were tailored to a particular cultural context or group. TBI = traumatic brain injury. ADHD = attention-deficit/hyperactivity disorder. ASD = autism spectrum disorder. CBT = cognitive behavioral therapy.

* Case studies were included for descriptive purposes but certain equity conditions (e.g., reporting sociocultural characteristics of sample) may not apply as case studies often alter the characteristics of their participants to ensure confidentiality.

**Table 3:** Intervention Components (N = 29)

Intervention name	Positive Parenting				Promoting Their Children’s Healthy Lifestyles				Managing chronic health condition	Separate child module
	C	PR	LS	P	Child sleep habits	Child eating habits	Child phys. activity	Child screen time		
I-InTERACT Express (51)	x	x	x	x						
RUPP (52)	x	x	x	x	x (opt)	x (opt)				
FCU4Health (33,34)	x	x	x		x	x	x	x	x (obesity)	
Grow (53)	x	x	x	x	x	x	x	x		
Parent training intervention (54)	x	x	x		x			x	x (anxiety)	
Family-based telemedicine intervention (55)		x	x			x	x	x		x (concurrent child sessions)
Enhanced ParentCorps (47)	x	x	x	x	x	x	x			x (concurrent child sessions)
PCIT-Health (56)	x	x	x	x	x	x		x		x (live coaching sessions)
More and Less parent group program (57)	x	x	x	x		x	x	x		
LEAP (58)	x	x	x	x	x		x	x	x (ADHD)	
Behavioral sleep intervention (59)		x	x		x	x				
PT-F (60,61)	x	x	x			x			x (ASD)	
Mindful M&M’s (62)	x	x	x	x	x	x		x	x (Disruptive Behavior Disorder)	x (child mindfulness program)
parent training program (63)	x		x	x	x		x	x		
Combined Sleep and Standard Behavioral Treatment (64)	x	x	x		x	x		x		x
HS+POPS+IYS (48)	x	x	x	x		x		x	x (obesity)	x (child obesity prevention program)
Parent-based sleep education (65)	x	x	x		x	x	x		x (sleep apnea, restless leg syndrome)	
Parent Management Training + Sleep Train Program (66)	x	x	x		x					
Hassle Free Mealtimes Triple P (67)	x	x	x			x				
Girls Growing in Wellness and Balance (68)	x	x	x			x				x (eating disorder prevention group)
CBT-based Bibliotherapy Plus Doll (69)	x	x	x		x					
Behavioral parent training (70)	x	x	x		x	x			x (ASD)	
DELFIN parenting program (71)	x		x						x (chronic illnesses)	
ENTREN-F program (72)	x					x	x		x (obesity)	x (child emotion regulation)
Cognitive-behavioral, skill-building intervention (73)		x	x			x	x			
SFSW intervention (74)	x	x	x							
Parent-Assisted CBT (75)		x			x					
CHIP-Family^a^ (46)							x		x (congenital heart disease)	x (CBT exercises)
Triple P (76)	x	x	x						x (diabetes)	
**Totals (out of 29)**	**24 (83%)**	**25 (86%)**	**26 (90%)**	**10 (34%)**	**16 (55%)**	**18 (62%)**	**11 (38%)**	**11 (38%)**	**12 (41%)**	**9 (31%)**

*Note*. It is worth noting that positive parenting and parent-targeted information for promoting children’s healthy lifestyles were often intertwined rather than mutually exclusive categories. C = communication-related skills, such as giving clear directions, active listening, open-ended questions, problem solving, and warmth. PR = positive reinforcement, such as praise, reward/incentive systems, and behavioral reinforcement strategies. LS = limit setting, such as enforcing house rules, use of logical consequences, planned ignoring, or time out. P = play strategies, such as child-directed play. Chronic health condition = intervention contains information on a chronic physical or mental health condition, such as diabetes, obesity, asthma, ASD, ADHD, or anxiety. Opt = content was optional for participants.

a Study focused on general parenting skills but did not describe any specific strategies.

**Table 4:** Summary of Effects Separated by Child Health Outcome

Outcome	Beneficial Effects	Null Effects	Adverse Effects	Total Effects
**Emotional health**	72 (51%)	68 (49%)	0 (0%)	140
Social functioning	6 (46%)	7 (54%)	0 (0%)	13
Emotional functioning	19 (44%)	24 (56%)	0 (0%)	43
Cognitive-behavioral functioning	47 (56%)	37 (44%)	0 (0%)	84
**Healthy lifestyle behaviors**	49 (47%)	55 (53%)	0 (0%)	104
Sleep	29 (57%)	22 (43%)	0 (0%)	51
Eating habits	8 (36%)	14 (64%)	0 (0%)	22
Physical activity	7 (33%)	14 (67%)	0 (0%)	21
Screen time	2 (33%)	4 (67%)	0 (0%)	6
General healthy lifestyle behaviors	3 (50%)	3 (50%)	0 (0%)	6
**Physical health-related indicators**	3 (15%)	17 (85%)	0 (0%)	20
Weight or BMI	1 (8%)	11 (92%)	0 (0%)	12
Disease symptom management	1 (50%)	1 (50%)	0 (0%)	2
Somatic concerns	1 (25%)	3 (75%)	0 (0%)	4
**Total Effects**	124	140	0 (0%)	264

*Note*. Pre-post intervention outcomes were synthesized and categorized by outcome domain: child emotional health (i.e., social, emotional, and cognitive-behavioral functioning) and child physical health, including healthy lifestyle behaviors (i.e., sleep, eating habits, physical activity, and screen time) and other physical health-related indicators (i.e., weight, BMI, disease symptom management, and somatic concerns). The three case studies were excluded from these calculations due to a lack of reliable significance testing (56,62,75).

**Table 5:** Summary of Type of Effects on Each Health Domain

Type of Effects	Study Totals (out of 27 studies)
*Dual effects* on child social-emotional and physical health	*n* = 16 (59%)
Single effects-child emotional health *only*	*n* = 2 (7%)
Single effects-child physical health *only*	*n* = 4 (15%)
No Effects	*n* = 5 (19%)

*Note*. Studies were coded as showing *no effect*, a *single effect*, or a *dual effect* based on whether they reported at least one significant improvement (*p* < .05) in each domain. For example, to be coded as showing a dual effect, the study needed to report at least one significant outcome in both emotional and physical health domains. The total sample size consisted of 27 studies, as case studies were excluded (*n* = 3). The FCU4Health intervention reported emotional and physical outcomes in two separate articles, but is listed only once in this table.

## Data Availability

The full search strategy for all databases and extraction and charting forms will be made available upon reasonable request. The full search strategy for PubMed and extraction template is included in the supplemental files.
